# Social representations of oncologic surgery for patients with cancer

**DOI:** 10.1590/0034-7167-2023-0273

**Published:** 2025-01-13

**Authors:** Rômulo Frutuoso Antunes, Rachel Verdan Dib, Raquel de Souza Ramos, Antonio Marcos Tosoli Gomes, Manassés Moura dos Santos, Margarida Maria Rocha Bernardes, Carolina Cristina Scrivano dos Santos, Karen Paula Damasceno dos Santos Souza

**Affiliations:** I Instituto Nacional de Câncer. Rio de Janeiro, Rio de Janeiro, Brazil; II Hospital Israelita Albert Einstein. São Paulo, São Paulo, Brazil; III Universidade do Estado do Rio de Janeiro. Rio de Janeiro, Rio de Janeiro, Brazil; IV Escola Superior de Guerra. Rio de Janeiro, Rio de Janeiro, Brazil

**Keywords:** Social Representation, Surgical Oncology, Oncology Nursing, Medical Oncology, Neoplasms, Representación Social, Oncología Quirúrgica, Enfermería Oncológica, Oncología Médica, Neoplasias

## Abstract

**Objectives:**

to analyze the social representations of patients with cancer regarding oncologic surgery.

**Methods:**

a qualitative study based on Social Representation Theory was conducted with 126 participants between October 2021 and May 2022 in a public hospital in Rio de Janeiro. A characterization questionnaire, free evocations of the inducing term “surgery”, and semi-structured interviews with 60 participants were applied. Data were analyzed using Microsoft Excel® and IRaMuTeQ.

**Results:**

the central core of the representation is composed of fear, cure, hope, and removing the disease. The analysis of interviews resulted in six classes that highlight the social changes caused by treatment as well as the need for a support network to cope with the surgical process.

**Final Considerations:**

the representations reflect fear and hope towards the procedure and the desire to remove the disease, thus translating the cure through surgery.

## RESUMEN

**Objetivos:**

analizar las representaciones sociales de los pacientes con cáncer respecto a la cirugía oncológica.

**Métodos:**

estudio cualitativo, basado en la Teoría de las Representaciones Sociales, realizado con 126 participantes entre octubre de 2021 y mayo de 2022 en un hospital público de Río de Janeiro. Se aplicó el cuestionario de caracterización de los sujetos, las evocaciones libres del término inductor“cirugía”y la entrevista semiestructurada con 60 participantes. Los datos fueron analizados utilizando Microsoft Excel® e IRaMuTeQ.

**Resultados:**

el núcleo central de la representación está formado por miedo, cura, esperanza y eliminación de la enfermedad. El análisis de las entrevistas originó seis clases que evidencian los cambios sociales provocados por el tratamiento, además de la necesidad de una red de apoyo para enfrentar el proceso quirúrgico.

**Consideraciones Finales:**

las representaciones reflejan el miedo y la esperanza ante el procedimiento y el deseo de eliminar la enfermedad, traduciendo así la cura a través de la cirugía.

## INTRODUCTION

Cancer is the name given to a group of more than 100 types of diseases that affect an individual’s body and can cause irreparable damage^(1)^. Approximately 70% of global deaths from neoplastic diseases occur in lowand middle-income countries, where late diagnosis and barriers to access to treatments increase mortality rates^(2)^. The *Instituto Nacional de Câncer* (INCA, National Cancer Institute), the agency that provides epidemiological information on cancer in Brazil, estimates that, for the 2023-2025 triennium, there will be a 12.6% increase in cancer cases in Brazil, accounting for approximately 704 thousand new cases of cancer when compared to the estimates for the 2020-2022 triennium^(3)^.

Exposure to risk factors increases the likelihood of developing the disease. However, changes depend on individual and collective lifestyles, the development of government actions and regulations, and the results of new research^(1)^. A cohort study with 1,277 patients revealed that 16.2% reported one or more social risks, with financial difficulties being the most common. Moreover, a higher proportion of patients with one or more social risks was women (n=123/207, 59.4%)^(4)^.

In Brazil, according to data from the Oncology Observatory, around 10,000 oncological surgeries are performed every year^(5)^. Currently, with the advent of new technologies applied in the field of health, treatment has become less aggressive compared to what it was a short time ago, when extensive and highly invasive surgeries were performed^(5)^.

Patient preparation is essential for a good recovery after surgery, since having to undergo surgery causes anxiety, fear, anguish, doubts, among other feelings regarding the procedure^(6)^. Another important aspect is changes in body image, such as asthenia, weight loss, and the presence of scars/mutilations that significantly impact body self-image^(7)^.

Research on social representations (SR) of cancer and its treatments^(8,9,10)^ reveals the diversity of feelings experienced by patients when faced with the disease, highlighting the importance of exploring these representations in their entirety. While stigma related to cancer persists, there are also positive aspects, such as treatment perception as a catalyst for changes in the disease, together with social and spiritual support^(9)^.

A literature review on SR of cancer^(11)^ emphasizes themes such as finitude, fear, physical changes, complexities of treatments and the relevance of a support network. These elements are fundamental to understanding patients’perceptions of their diagnosis and how they plan their routine to deal with treatment. Therefore, a holistic and patient-centered approach is essential to deal with the challenges associated with the disease and therapy, providing information and filling knowledge gaps to facilitate their participation in the therapeutic process.

Thus, the study is justified by the fact that cancer has great importance in public health and in social life of individuals living with the disease, as it directly interferes with the way of life and social relationships^(12)^. This study is relevant because of the impact of surgery on the way of life and the way of conducting life in the different social scenarios of people who undergo the procedure, as well as promoting information that adds to preand post-procedure nursing care and public health policies, to offer the necessary subsidies for rehabilitation to the new living condition.

## OBJECTIVES

To analyze the SR of patients with cancer regarding oncological surgery.

## METHODS

### Ethical aspects

The study followed the standards and guidelines for conducting studies involving human beings of Resolution 466/12 of *Conselho Nacional de Saúde* (CNS, Brazilian National Health Council)^(13)^. The legal basis for conducting this study is supported by the Research Ethics Committee (REC) of the Municipal Health Department of Rio de Janeiro, whose opinion is attached to this submission. Participants were provided with two copies of the Informed Consent Form (ICF) in writing.

### Study design and theoretical framework

This is a descriptive study with a qualitative approach, which followed the criteria present in the COnsolidated criteria for REporting Qualitative research (COREQ), based on Social Representation Theory (SRT), guided by Serge Moscovici (1978)^(14)^, through the structural analysis of Abric (2000)^(15)^. Through the study of SR, it is possible to understand the relationship of common sense about an object that interacts in individuals’social and cognitive adaptation in the face of everyday reality and social and ideological characteristics^(14,15,16)^. Furthermore, the growing contribution of SRT in studies produced in the field of nursing makes it unique in psychosocial phenomena by exploring health objects as subject to interpretation^(17)^. In this case, users’social and cognitive perception regarding surgical treatment is included.

The Central Nucleus Theory, proposed by Abric (2000), suggests that the SR structure occurs around a central nucleus (CN), which is made up of elements that reveal the collective SR, being more resistant to changes, thus demonstrating continuity of the representation^(15)^. The other elements, the peripheral ones – 1^st^ and 2^nd^ periphery and contrast zone (CZ) -, are located around the possible CN and are ordered by it^(15,18)^.

### Location, population and inclusion and exclusion criteria

The study was conducted at a federal public hospital, a reference in oncology, located in the city of Rio de Janeiro, with 126 participants. Patients with a diagnosis of malignant neoplasia confirmed by biopsy, clinical or surgical, aged 18 years or older and who had clinical conditions to participate in the study were included. People with Eastern Cooperative Oncology Group (ECOG) less than or equal to 3 and patients undergoing end-of-life care were excluded.

### Study protocol, data collection and data organization

Data collection took place from October 2021 to May 2022. Non-probabilistic convenience sampling was adopted, chosen based on the number of patients undergoing treatment. To collect sociodemographic and clinical data, a questionnaire to characterize the subjects was applied to 126 participants, containing the following data: age; sex; marital status; education; place of residence; religion; time since diagnosis; whether there is a history of cancer in the family, how many people and degree of kinship (if the answer is yes, whether patients have undergone treatment previously and, if the answer is yes, which treatment(s)).

The free evocation form, used to explore the SR structure, asked participants to mention the first five words or expressions that came to mind when hearing the inducing term “surgery”, which were recorded in the order in which they were mentioned. It is important to note that all free evocation forms were completed by the researcher during data collection, without offering participants time to reflect on the evocations, which may impact the proposed methodology. All 126 study participants completed both the characterization questionnaire and the free evocation form. All responses to the subject characterization questionnaire as well as the free evocations were transcribed into Microsoft Excel^®^ by the researcher.

The interviews were conducted with 60 patients, conveniently selected from the 126 participants in the previous stages. Selection was based on patients’ willingness to share more details about their perceptions and experiences regarding cancer surgery. The interviews were conducted at the patients’ bedside, with care taken to ensure their comfort and privacy. During the interviews, a semi-structured script was used addressing three thematic chunks: 1. SR of cancer surgery; 2. Perceptions of life before and after surgery; and 3. Care relationships with the health team and the service. Each interview lasted an average of 30 minutes.

### Data analysis

To analyze the collected data, Microsoft Excel^®^ was used to analyze participant sociodemographic profile, and the *Interface de R pour les Analyses Multidimensionnelles de Textes et de Questionnaires* (IRaMuTeQ) was used to perform the lexical analysis of the evocations and interviews. Furthermore, the Reinert Method was used to analyze the interviews. Subjects were coded by number (patient 001, patient 002, and so on), sex (female and male), age (under or equal to 52 years and over 52 years). Additionally, the words that were most significant (p < 0.0001) for each class distributed by the Descending Hierarchical Classification (DHC) dendrogram will be described and marked with the frequency value in the class (f ) and chi-square (x^2^). This procedure aims to provide a deeper understanding of the results obtained. The Elementary Context Units (ECUs) are textual segments gathered from the text material. Through them, it is possible to understand the speeches elaborated by subjects in response to the questions asked in this study^(19)^.

## RESULTS

The sociodemographic characterization of the 126 participants shows that the majority are male (n=74, 58.73%). The predominant age group is over 52 years old (n=68, 53.97%). Regarding marital status, half declared being married (n=63, 50%). Concerning educational level, less than half had completed high school (n=48, 38.10%), whereas another large group had incomplete elementary school (n=35, 27.78%). Most participants live in the Metropolitan Region of Rio de Janeiro (n=100, 79.37%). As for religion, the majority declared following the Catholic and Evangelical religions (n=52, 41.27%, respectively). The majority had been diagnosed with cancer less than one year ago (n=59, 46.83%), and more than half had not undergone cancer treatment previously (n=67, 53.17%). In relation to family history of cancer, a large proportion stated that they had had cases of cancer in a relative, regardless of the degree of kinship (n=85, 67.46%).

According to the results of all participants’free evocations, with the help of IRaMuTeQ, the following parameters were adopted: a minimum word frequency of 5, which means that terms with a frequency lower than this value were excluded from the analysis; an average frequency of 13.26; and an average of the average orders of evocation (AOE) equal to 2.7. The AOE is related to the average position of each term evoked in the analyzed *corpus*, indicating that the lower the AOE value, the more readily it was evoked by the subjects in the first instance to the inducing element “surgery”. From the aforementioned parameters, the software generated the Four-Quadrant Chart, presented in Chart 1, with the contents and their organization.

**Chart 1:** Four-Quadrant Chart for the inducing term “surgery” for people diagnosed with cancer treated at a High Complexity Oncology Center, Rio de Janeiro, Rio de Janeiro, Brazil, 2022 (N=126)

OME	≤ 2,7			>2,7		
Med. Freq.	Evoked term	Freq.	AOE	Evoked term	Freq.	AOE
≥13.26	Fear	47	2.1			
	Cure	29	2.7	Treatment	17	2.9
	Hope	19	2.7	Anesthesia	16	3.1
	Removes disease	16	2.1			
< 13.26				God	12	3.4
	Pain	13	2.5	Scar	11	3.2
	Cut	12	2.3	Nervous	11	3.0
	Necessary	10	1.8	Calm	11	2.9
	Be well	9	2.3	Doctor	10	3.0
	Health	9	2.7	Recovery	9	3.1
	Anxiety	7	2.7	Good	9	3.3
	Improvement	6	2.5	Life	8	3.5
				Fear	8	3.1
				Trust	6	3.8

*AOE – average order of evocation.*

It can be observed in this study that the possible CN is formed by “fear”, “cure”, “hope” and “removes disease”. The lexicon “fear” was the most evoked term (47) and most readily spoken by the subjects (AOE = 2.1) to the inducing term “surgery”, and this reflects the negative feeling that patients feel towards surgical treatment for cancer. “Cure” was the second most evoked term (29) by subjects, with AOE 2.7, i.e., participants see surgery as a procedure that will bring about a cure for cancer. It is an element with positive content that generates motivation and hope for the procedure to be performed.

“Hope”is the third most frequently evoked element (19), with an AOE of 2.7, reinforcing the desire for a cure, because, despite the fear, they have hope that, through surgery, they will be free from cancer. This term refers to the imagery dimension of surgical treatment as a life-changing factor. Finally, the expression “removes disease” is the element that has the lowest frequency (16) of evocation in the quadrant, but has the lowest AOE (2.1), since surgery removes the disease and, consequently, cures the disease. It is an attitudinal representative element that reflects the result of surgery.

The inclusion of terms “cure” and “removes disease” in the CN reveals participants’ complex SR about surgical treatment for cancer. “Cure” denotes the deep hope for a complete recovery and definitive elimination of the disease, reflecting the search for a positive and lasting result. On the other hand, “removes disease” highlights the perception of surgery as a practical and concrete process of physically removing cancer from the body, thus resulting in the eradication of the disease. These two terms, although different in their connotation, share a common goal of overcoming cancer, representing both the symbolic dimension of hope and the pragmatic dimension of surgical intervention. The inclusion of both in the study’s CN illustrates the multiplicity of meanings attributed to cancer surgery by patients, showing the complexity and richness of their SR on the topic.

The first periphery is formed by “treatment” and “anesthesia”. These are elements linked to the surgical therapeutic proposal and come from the cognitive dimension of what surgery is and its instruments of performance, since, for patients, anesthesia is one of the stages of surgical treatment. These are elements with a high frequency of evocation, but high AOE, which justifies their not participating in the possible CN of the representation.

The second periphery is made up of elements“God”,“scar”,“nervous”,“calm”,“doctor”,“recovery”,“good”,“life”,“fear”and“trust”. The term“God”was the most evoked within this quadrant, representing spiritual support. The triad“scar”,“nervous”,“calm”represented the second most evoked terms, whereas “calm” had the lowest AOE within the periphery, being elements of cognitive and affective/attitudinal dimensions. The term“doctor”symbolizes the professional who is in charge of treatment and a vehicle for the realization of the process. The terms “recovery”and “life”belong to the imagery dimension, whereas“good”,“fear”and“trust”belong to the affective/ attitudinal dimension, with“trust”being the term with the lowest frequency of evocation and the highest AOE.

This study’s CZ is formed by elements “pain”, “cut”, “necessary”, “be well”,“health”,“anxiety”and“improvement”. The terms present in this quadrant reinforce the idea present in the CN, since fear reflects anxiety, pain, the cut and the scar from the procedure. However, the term “necessary” has the lowest AOE (1.8) of the entire chart, i.e., patients understand that surgery is a treatment to cure cancer and is effective in recovering health (“improvement”) and being well.

**Figure 1:**
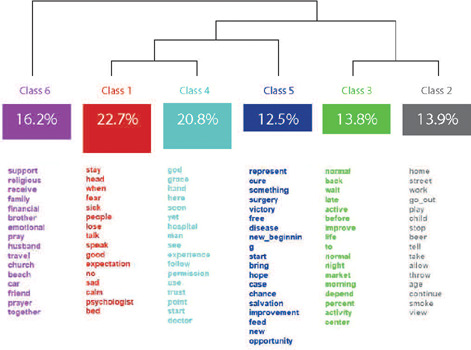
Hierarchical Descending Classification Dendrogram by semantic contente, Rio de Janeiro, Rio de Janeiro, Brazil, 2022 (n= 60)

According to the analysis of interviews, through the Reinert Method, the DHC dendrogram was created, with six classes, from the 726 text segments, with 92.13% utilization of the analyzed *corpus*. Initially, a first division occurred, in which an axis was formed with class 6 (16.2%) and another axis with the other segmental classes. In a second division, an axis was formed with classes 3 (13.8%) and 2 (13.9%), on one side, and another axis with class 5 (12.5%), on the other side. In a third division, classes 1 (22.7%) and 4 (20.8%) were generated, as shown in Figure 1.

From the analysis of the aforementioned classes, six classes were identified, which will be presented below.

### Class 1 – Impact of cancer diagnosis and surgical treatment

Class 1 is the largest in the *corpus*, consisting of 165 ECUs, accounting for 22.7% of the *corpus*. The elements that stood out the most in this class are stay (f71, x^2^=47.5), head (f19, x^2^=38.93), fear (f=25, x^2^= 23.95), sick (f=8, x^2^=22.71), lose (f=10, x^2^=19.28), talk (f=8, x^2^=18.94) and sad (f=9, x^2^=16.3).

The elements reflect the impact of diagnosis and surgery on patients’ lives as well as the feelings they experience at this time. The fear of the unknown, of what might happen during the surgery, and of the invasiveness that the procedure demands sounds like an avalanche of feelings in patients. It is also worth noting that the imagery dimension of treatment may sometimes not correspond to the expected result of treatment, which also generates sadness and frustration.

*I’ll tell you the truth, I felt scared. [...] fear, anxiety, nervousness, it’s a mix of feelings that we can’t really explain.* (Patient 034, male, 52 or older)

*For me, surgery is scary. We feel scared, but at the same time we feel obligated to do it, because it’s a process of healing and hope.* (Patient 036, female, 52 or older)

*For me, the surgery was a hope, but before I had a lot of faith, a lot of hope, but after I did it, I got really depressed, I got really frustrated, because it’s not what I imagined in my head* [...]. (Patient 046, female, 52 or older)

### Class 2 – Participants’ lifestyle habits and social routine before cancer diagnosis

Class 2 is formed by 101 ECUs, accounting for 13.9% of the entire *corpus*. The terms that stood out the most are house (f=27, x^2^= 88.06), street (f=11, x^2^=69.12), work (f=28, x^2^= 55.27) and go_out (f=19, x^2^= 50.05). This group of words corresponds to participants’ lifestyle habits and social routine before cancer diagnosis. The terms play, child and street refer to memories and past social life, while beer, smoking, working and going out refer to daily habits.

*Before I was diagnosed with cancer, my life was wild. I smoked, drank, did everything. I stayed up all night, all night, traveled too much, worked too much, everything, everything too much.* (Patient 018, male, 52 years or older)

*It was a normal life. I played in the street, until after I was diagnosed, I did things, I tried to lead a normal life,* [...], *throw a ball, play like a child,* [...]. *These things, I had to back off a little, stop for a while.* (Patient 024, male, less than or equal to 52 years old)

### Class 3 – Daily practices and their relationship with surgical treatment: redefining the new reality

Class 3 is constructed by 100 ECUs, accounting for 13.8% of the analyzed *corpus*. The words that stand out in the class are normal (f=43, x^2^= 114.14), back (f=24, x^2^=54.84), wait (f=21, x^2^= 45.46), before (f=22, x^2^=32.09) and life (f=45, x^2^=28.64). It is noteworthy the very close parity with class 2, however this mentions daily practices and the desire to return to normal daily life, as it was before undergoing the procedure and the diagnosis, also highlighting the desire to be independent, a facet masked after undergoing the surgical process.

*Health, life, the surgery brought me health and life*. (Patient 013, female, 52 years or older)

*That I can work, travel to see my family, take care of my home, my life as it was before, not depend on others to do things for me.* (Patient 015, female, 52 years or younger)

*And with the surgery, it will solve a lot of things in my life, it will give me more energy. It will get me back into the job market. That was also preventing me from going back to work.* (Patient 032, female, 52 years or younger)

Patients need to readapt to their new living conditions in a way that brings them as close as possible to their previous normality. Social readjustment and the desire for recovery help to keep them more optimistic about their new living conditions.

### Class 4 – Practical dimension of surgery, routine oncological treatment and the spiritual dimension

Class 4 is composed of 151 ECUs, accounting for 20.8% of the *corpus*. The terms that stand out in this class are God (f=59, x^2^=89.84), grace (f=15, x^2^=36.68), hand (f=13, x^2^=29.63), here (f=40, x^2^=25.96), hospital (f=9, x^2^=18.85), experience (f=4, x^2^=15.32) and permission (f=4, x^2^=15.32).

According to the elements that make up this class, the practical dimension of surgery stands out in patients’ discourses through cognems, which express the routine of oncological treatment and the spiritual dimension in this new scenario that presents itself. The terms reflect the feelings present at the time of hospitalization, which translates into the moments of pre-, transand immediate post-operative care. On the other hand, there are elements that reflect faith in a Being that transcends the carnal planes, such as God.

*Everything that happens will be God’s will, God’s permission.* (Patient 007, male, 52 or younger)

[...] *the first time I met the doctor, I felt God’s presence in him.* (Patient 035, female, 52 or younger)

### Class 5 – Feelings and anxieties regarding surgery: oncological cure

Class 5 is the smallest in the *corpus*, consisting of 91 ECUs, accounting for 12.5% of the *corpus*. This group reflects the feeling and desire placed on the success of surgical treatment to continue a life free from disease, as can be seen through the elements cure (f=21, x^2^=103.65), victory (f=7, x^2^=49.32), free (f=7, x^2^=41.47), disease (f=19, x^2^=40.13), new_beginning (f=5, x^2^=35.13), hope (f=12, x^2^=31.78) and salvation (f=4, x^2^=28.07).

*It is the possibility of me being free from cancer and returning to a normal life. The cure.* (Patient 053, male, 52 or older)

*In my case, the surgery was a salvation, for me it was salvation, a new beginning and a great victory*. (Patient 013, female, 52 or older)

*It represents getting rid of a problem, it represents victory.* (Patient 049, male, 52 or younger)

Based on the discourse of the interviews, it is possible to contextualize the elements present in the representation’s CN, in which the SR of surgery for cancer patients revolves around the hope of eliminating the disease and, consequently, being cured through surgical treatment to the point that they feel afraid of surgery and the setbacks that may arise in the postoperative period.

### Class 6 – Patient support network for oncological surgery

Class 6 is composed of 118 ECUs, accounting for 16.2% of the *corpus*, with the main words support (f=37, x^2^=163.57), religious (f=23, x^2^=122.39), receive (f=29, x^2^=106.27), family (f=36, x^2^=105.83) and financial (f=20, x^2^=99.12). Analyzing the lexical content of the interviews that form class 6, it is clear that this reflects the social, spiritual, mental and financial support that patients receive before surgery. These characteristics are perceived through the following discourses:

*From my family, yes, a lot of support, my family was everything, they were my foundation and so were my friends.* (Patient 003, male, 52 or younger)

*I received a lot of support from my family, many friends. The psychologist here also helped me a lot. The doctors also gave me legal support.* (Patient 006, female, 52 or younger)

It is observed that social, spiritual and professional ties feed back into the foundations that supported surgical treatment, as can also be confirmed through prototypical analysis (Chart 1). The importance of the presence of God-family-professional is highlighted to support the life-threatening diagnosis, ensure confidence in the proposed treatment and build positive feelings in the face of the changes caused by the disease.

## DISCUSSION

The SR of oncological surgery for cancer patients is organized around three dimensions: practical, which includes habits and actions before diagnosis and in relation to surgery; affective, portrayed here in a continuum with two poles, with the first being fear and the second, at the other extreme, being hope; and imagery, in which healing, removal of disease and treatment are interrelated, forming, together, an image of oncological surgery that is embodied in a specific intervention that resolves specific and well-defined changes.

It is worth noting that anesthesia is also part of the imagery dimension, due to an ambiguity in its symbolic construction. On the one hand, it is essential for performing surgery. On the other hand, it is present in daily conversations that contain negative stories and experiences related to it, such as death after being sedated (sleeping and not waking up) and sequels as consequences of its use. It is important to point out, in the set of results, the group’s social identity that is shown in the presence of a support network as well as a relational aspect of patients with the dimension of the sacred.

Fear, anxiety and nervousness are feelings that are very present in patients because surgery is an invasive procedure that requires a lot of care in the preand post-operative period^(20,21)^. Hospitalization is often a new scenario that generates many negative feelings as well as the lack of information or misunderstanding of how surgery will be performed^(22)^. A study^(23)^ carried out with 25 patients undergoing surgery shows that anxiety is a very common feeling in patients, which makes them experience tingling sensations, fear of what might happen and of death, nervousness, among others.

Anticipatory anxiety is a clinical concern rather than a concern related to the surgical procedure itself. These emotional reactions suggest that individuals construct collective mental representations about events and situations, influenced by social and cultural factors. A study conducted with 3,087 surgical patients undergoing any type of anesthesia and surgery indicated that 40% of patients were anxious in the preoperative period^(24)^. Another study with 109 patients undergoing brain tumor biopsy or craniotomy with tumor resection highlighted that 30% of patients presented clinical levels of anxiety before the procedure, compared with 20% in a group of patients undergoing elective spine surgery^(25)^.

Another experience experienced by patients is the changes in body parts caused by surgeries, which generate many losses, including self-esteem, social interaction and autonomy. Therefore, it is clear that the therapy harms patients in several physical, social and mental aspects^(26,27,28)^. According to the research by Peixoto *et al*.^(29)^ with 56 patients with stoma in the late postoperative period, it was shown that 48.2% of participants presented complications related to stoma. Furthermore, according to assessment of adaptation to the stoma, in the positive acceptance domain, people had an average score, i.e., the majority did not adapt to the stoma. This reflects the fear, the scar, the cut, the reified knowledge of patients regarding surgery as well as post-surgery dissatisfaction expressed by participant 046, in class 1, of DHC analysis.

A study conducted with 41 patients diagnosed with oral cavity neoplasia revealed that more than 80% of patients received surgical treatments and that, after tumor excision, patients presented permanent deformities with a direct impact on their overall quality of life (OQoL). Males presented the worst OQoL in the social sphere, whereas females presented the worst in the physical scale^(30)^. Furthermore, the same study highlights that the word “fear” was linked to the terms “sad” and “dying”, thus reflecting the awareness of a life-threatening disease^(30)^, but in this study, fear was more linked to the procedure itself than to cancer diagnosis.

Surgery is seen as a form of treatment for patients and, through it, it is possible to achieve a cure. In the work of Antunes *et al*.^(20)^ with 111 participants, the term “treatment” is present in the possible CN of the representation, while in this study, the same element is found in the 1st periphery, that is, this reinforces that, at some point, this term will be a central element of the representation, reinforcing the cognitive dimension regarding surgery. Furthermore, the term “cure” is found in the possible CN of the representation, thus reaffirming treatment as an intermediary of cure.

The study showed that patients see cancer as a normal and difficult disease, but they believe in treatment and a cure through the available resources, even knowing that it will be a difficult process. Furthermore, the results indicate that the cure will be achieved through the intervention of God and treatment, whether associated or not^(31)^. Research on the SR of cancer revealed that the element “hope” was the most readily evoked by subjects in the second periphery, thus reflecting confidence and optimism in treatment in search of a cure for cancer^(31)^.

Spirituality/religiosity is an important coping mechanism when faced with surgical procedures^(32)^. In the second periphery, the presence of God, doctor and trust stands out. This scenario focuses on the path towards a cure for cancer through two aspects that are not mutually exclusive: scientific knowledge represented by the doctor; and faith in a higher entity, manifested by religiosity (God). Research shows that the moment of oncological diagnosis has been one of the most traumatic moments from the psychological and existential point of view of patients and their families, this being a fundamental dimension in coping with the pathology, in which faith is the central axis and guide of the process of disease recovery, hope and cure. Furthermore, religiosity is a fundamental resource when faced with hospitalization and illness, becoming an important tool for dealing with pain, anguish, fear and other negative feelings linked to this process^(22,32,33,34)^.

In addition to spiritual support, patients rely on family support during the cancer diagnosis, thus revealing a fundamental and persistent tool throughout the treatment process of the disease. In the study by Wakiuchi *et al*.^(31)^, this support appears through the term “family”, in the second periphery. In this study, support is seen through DHC analysis, in class 6. A study by Fischer and Seibaek^(35)^ reveals that, in addition to family support, the social group, such as neighbors and people suffering from the same disease, proved to be a valuable mechanism for patients.

Some patients reported a desire to return to normal. In other words, this feeling expresses the desire to live as close to what they lived before undergoing the surgical procedure as possible, such as working, not having many restrictions on their way of life, and giving new meaning to some moments that were not lived. In a study on the experience of people living with advanced stage cancer, the authors state that“normality”is the adjustment to the new reality, corresponding to the life one had before having the disease as well as developing a new relationship with the finite being and death^(36)^.

Cancer treatment causes major changes in the daily lives of people with the disease, such as withdrawal from the job market, social life and feeling active. A systematic review with metaanalysis of observational studies^(37)^ identified that breast cancer surgery is associated with higher unemployment rates, 26 studies (n=46,927 patients) associated 127 variables with unemployment after the procedure. Among the factors that predispose to withdrawal from the labor market, the high psychological and physical demands of work stand out, associated with other demands after surgery, such as rehabilitation, combination of chemotherapy and radiotherapy, constant visits to the hospital and the care required by mastectomy, generating employment support or accommodation in flexible working hours, paid leave and/or modifications of work tasks^(37)^.

### Study limitations

One limitation of this study is the fact that some patients were approached in the immediate and mediate postoperative period. Such conditions affect the perception of life after surgery in the extra-hospital environment, since they were still hospitalized and under the care of a nursing team. In this context, the possibility arises for new studies that address the representations of surgery in the late postoperative period, in which patients have already had contact with its reality in everyday life.

### Contributions to nursing, health or public policy

Thus, it is believed that, through this study, health professionals will be able to base their care to meet the demands and representations listed by subjects when faced with such a procedure, with a view to reducing the stress caused by the procedure. It is also believed that innovation and care promotion will be achieved through the results of this research, by understanding and knowing the SR of surgery for patients, because, by revealing such representations, it will be possible to access and explore the attitudes, values, thoughts and practices of how a group behaves when faced with disease and treatment, promoting more holistic and humanized care.

## FINAL CONSIDERATIONS

The feelings regarding surgery are ambivalent, due to the time that patients feel distress, fear of surgery and fear of post-procedure setbacks. Additionally, trust in God and the doctor is identified; with this, some users feel calm and confident, knowing that the surgery will be optimistic, enabling the cure or absence of disease.

Based on the prototypical analysis and the discourses, it is possible to describe a situation regarding the importance of healing as an expected result after surgery, predominantly assessed as removal of disease. These are elements with positive content, except for“fear”, which represents negative factors of the surgery. Based on the contents of the possible CN, it is possible to identify the presence of affective-attitudinal and cognitive dimensions that compose the SR.

Therefore, multidisciplinary care for people living with cancer undergoing surgical treatment needs to be reviewed, considering the SR of the group where the term “fear” is most frequently mentioned, in addition to the idea of a cure through removal of disease. In view of this, preoperative consultations are extremely important, in order to clarify any doubts about the procedure as well as offering support to patients to minimize negative feelings about the procedure.
