# Supplementary material for: Social representations of oncologic surgery for patients with cancer
Source: Rev Bras Enferm. 2025 Jan 13;77(6):e20230273. doi: 10.1590/0034-7167-2023-0273 (PMC11731848; doi:10.1590/0034-7167-2023-0273)
Supplement: Translation

# Translation: Representações sociais da cirurgia oncológica para o paciente com câncer

**DOI:** 10.1590/0034-7167-2023-0273pt

**Authors:** Rômulo Frutuoso Antunes, Rachel Verdan Dib, Raquel de Souza Ramos, Antonio Marcos Tosoli Gomes, Manassés Moura dos Santos, Margarida Maria Rocha Bernardes, Carolina Cristina Scrivano dos Santos, Karen Paula Damasceno dos Santos Souza

**Affiliations:** I Instituto Nacional de Câncer. Rio de Janeiro, Rio de Janeiro, Brasil; II Hospital Israelita Albert Einstein. São Paulo, São Paulo, Brasil; III Universidade do Estado do Rio de Janeiro. Rio de Janeiro, Rio de Janeiro, Brasil; IV Escola Superior de Guerra. Rio de Janeiro, Rio de Janeiro, Brasil

**Keywords:** Representações Sociais, Oncologia Cirúrgica, Enfermagem Oncológica, Oncologia, Neoplasias

## Abstract

**Objetivos:**

analisar as representações sociais dos pacientes com câncer a respeito da cirurgia oncológica.

**Métodos:**

estudo qualitativo, fundamentado na Teoria das Representações Sociais, realizado com 126 participantes entre outubro de 2021 e maio de 2022 em um hospital público no Rio de Janeiro. Aplicaram-se o questionário de caracterização dos sujeitos, as evocações livres do termo indutor“cirurgia”e a entrevista semiestruturada com 60 participantes. Os dados foram analisados utilizando *Microsoft Excel®* e IRaMuTeQ.

**Resultados:**

o núcleo central da representação é formado por medo, cura, esperança e retira a doença. A análise das entrevistas originou seis classes, evidenciando as modificações sociais provocadas pelo tratamento, além da necessidade da rede de apoio para lidar com o processo cirúrgico.

**Considerações Finais:**

as representações refletem o medo e a esperança perante o procedimento e o desejo da retirada da doença, traduzindo, assim, a cura através da cirurgia.

## INTRODUÇÃO

O câncer é o nome dado ao conjunto de mais de 100 tipos de doenças que afetam o organismo do indivíduo, podendo causar danos irreparáveis^(1)^. Aproximadamente 70% das mortes globais por doenças neoplásicas ocorrem em países de baixa e média renda, onde o diagnóstico tardio e as barreiras de acesso aos tratamentos aumentam as taxas de mortalidade^(2)^. O Instituto Nacional de Câncer (INCA), órgão que disponibiliza informações epidemiológicas relativas ao câncer no Brasil, estima que, para o triênio 2023-2025, ocorra um aumento de 12,6% de casos de câncer no Brasil, representando cerca de 704 mil casos novos de câncer, quando comparado às estimativas do triênio 2020-2022^(3)^.

A exposição aos fatores de risco aumenta a probabilidade de desenvolver a doença. No entanto, as mudanças dependem dos estilos de vida individuais e coletivos, do desenvolvimento de ações e regulamentações governamentais, e dos resultados de novas pesquisas^(1)^. Estudo de coorte com 1.277 pacientes revela que 16,2% relataram um ou mais riscos sociais, sendo a dificuldade financeira o mais comum. Além disso, uma proporção maior de pacientes com um ou mais riscos sociais incluía mulheres (n=123/207, 59,4%)^(4)^.

No Brasil, de acordo com os dados do Observatório de Oncologia, são realizadas em torno de 10 mil cirurgias oncológicas todos os anos^(5)^. Atualmente, com o advento das novas tecnologias aplicadas no campo da saúde, o tratamento se tornou menos agressivo em comparação com o que era há pouco tempo, quando eram realizadas cirurgias de grande extensão e muito invasivas^(5)^.

O preparo do paciente é fundamental para que se possa ter uma boa recuperação no pós-operatório, haja vista que o fato de ter que realizar uma cirurgia causa ansiedade, medo, angústias, dúvidas, entre outros sentimentos diante do procedimento^(6)^. Outro aspecto importante são as alterações na imagem corporal, como astenia, emagrecimento e presença de cicatrizes/mutilações, que impactam significativamente a autoimagem corporal^(7)^.

Pesquisas sobre as representações sociais (RS) do câncer e seus tratamentos^(8,9,10)^ revelam a diversidade de sentimentos vivenciados pelos pacientes diante da doença, sublinhando a importância de explorar essas representações em sua plenitude. Enquanto o estigma relacionado ao câncer persiste, há também aspectos positivos, como a percepção do tratamento como catalisador de mudanças na doença, juntamente com o apoio social e espiritual^(9)^.

Revisão da literatura sobre as RS do câncer^(11)^ enfatiza temas como finitude, medo, alterações físicas, complexidades dos tratamentos e a relevância da rede de apoio. Esses elementos são fundamentais para compreender as percepções dos pacientes diante do diagnóstico e como eles planejam sua rotina para lidar com o tratamento. Portanto, uma abordagem holística e centrada no paciente é fundamental para lidar com os desafios associados à doença e à terapia, fornecendo informações e preenchendo lacunas de conhecimento para facilitar sua participação no processo terapêutico.

Desse modo, justifica-se o estudo pelo fato de o câncer ter grande importância na saúde pública e na vida social dos indivíduos que vivem com a doença, pois interfere diretamente no modo de viver e nas relações sociais^(12)^. A relevância do estudo se dá devido ao impacto da cirurgia no modo de viver e na forma de conduzir a vida nos diferentes cenários sociais das pessoas que passam pelo procedimento, bem como fomentar informações que agregam nos cuidados de enfermagem pré e pós-procedimento e nas políticas públicas de saúde, para oferecer subsídios necessários para reabilitação à nova condição de vida.

## OBJETIVOS

Analisar as RS dos pacientes com câncer a respeito da cirurgia oncológica.

## MÉTODOS

### Aspectos éticos

Foram respeitadas as normas e diretrizes para a realização de estudos envolvendo seres humanos da Resolução nº 466/12 do Conselho Nacional de Saúde (CNS)^(13)^. A base legal para a realização do presente estudo está apoiada pelo Comitê de Ética em Pesquisa (CEP) da Secretaria Municipal de Saúde do Rio de Janeiro, cujo parecer está anexado à presente submissão. Foi disponibilizado aos participantes o Termo de Consentimento Livre e Esclarecido (TCLE), por escrito, em duas vias.

### Tipo de estudo e referencial teórico

Trata-se de estudo descritivo com abordagem qualitativa, que seguiu os critérios presentes no *COnsolidated criteria for REporting Qualitative research* (COREQ), fundamentado na Teoria das Representações Sociais (TRS), orientada por Serge Moscovici (1978)^(14)^, através da análise estrutural de Abric (2000)^(15)^. Através do estudo das RS, é possível entender a relação do senso comum sobre um objeto que interage na adaptação social e cognitiva dos indivíduos diante da realidade do cotidiano e das características sociais e ideológicas^(14,15,16)^. Além disso, a contribuição crescente da TRS nos estudos produzidos na área da enfermagem o torna singular nos fenômenos psicossociais, por explorar objetos da saúde como passíveis de interpretação^(17)^. Nesse caso, insere-se a percepção social e cognitiva dos usuários a respeito do tratamento cirúrgico.

A Teoria do Núcleo Central, proposta por Abric (2000), sugere que a estrutura da RS ocorra em torno de um núcleo central (NC), constituído por elementos que revelam a RS do coletivo, sendo mais resistente às mudanças, demonstrando, assim, a continuidade da representação^(15)^. Já os demais elementos, os periféricos – 1ª e 2ª periferia e zona de contraste (ZC) -, localizam-se em torno do possível NC e são ordenados por ele^(15,18)^.

### Local, população e critérios de inclusão e exclusão

A pesquisa foi realizada em um hospital público federal, referência em oncologia, localizado na cidade do Rio de Janeiro, com 126 participantes. Foram incluídos pacientes com diagnóstico de neoplasia maligna confirmado por biópsia, clínicos ou cirúrgicos, com idade igual ou superior a 18 anos e que tivessem condições clínicas para participação no estudo. Foram excluídas pessoas com *Eastern Cooperative Oncology Group* (ECOG) menor ou igual a 3 e pacientes em cuidados de fim de vida.

### Protocolo do estudo, coleta de dados e organização dos dados

A coleta ocorreu no período de outubro de 2021 a maio de 2022. Foi adotada a amostragem do tipo não probabilística, de conveniência, escolhida a partir da quantidade de pacientes em tratamento. Para coleta de dados sociodemográficos e clínicos, aplicou-se um questionário de caracterização dos sujeitos com 126 participantes contendo os dados: idade; sexo; estado civil; escolaridade; local de moradia; religião; tempo de diagnóstico; se apresenta histórico de câncer na família, quantas pessoas e grau de parentesco (caso a resposta for positiva, se o paciente fez tratamento anteriormente e, sendo a resposta positiva, qual(is) tratamento(s)).

O formulário de evocações livres, usado para explorar a estrutura das RS, foi solicitado aos participantes que mencionassem as primeiras cinco palavras ou expressões que lhes viessem à mente ao ouvirem o termo indutor “cirurgia”, as quais foram registradas na ordem em que foram mencionadas. É importante observar que todos os formulários de evocações livres foram preenchidos pelo pesquisador durante a coleta de dados, sem oferecer aos participantes tempo para reflexão sobre as evocações, o que pode impactar a metodologia proposta. Todos os 126 participantes do estudo completaram tanto o questionário de caracterização quanto o formulário de evocações livres. Todas as respostas do questionário de caracterização dos sujeitos, bem como das evocações livres, foram transcritas no *software Microsoft Excel^®^* pelo pesquisador.

As entrevistas foram conduzidas com 60 pacientes, selecionados de maneira conveniente entre os 126 participantes das etapas anteriores. A escolha baseou-se na disposição dos pacientes em compartilhar mais detalhes sobre suas percepções e experiências em relação à cirurgia oncológica. As entrevistas foram realizadas junto ao leito do paciente, com cuidado para garantir seu conforto e privacidade. Durante as entrevistas, utilizou-se um roteiro semiestruturado abordando três blocos temáticos: 1. RS sobre a cirurgia oncológica; 2. Percepções da vida antes e depois da cirurgia; e 3. Relações de cuidado com a equipe de saúde e o serviço. Cada entrevista teve em média 30 minutos de duração.

### Análise dos dados

Para a análise dos dados coletados, utilizaram-se o *Microsoft Excel^®^*, para análise do perfil sociodemográfico dos participantes, e o *software Interface de R pour les Analyses Multidimensionnelles de Textes et de Questionnaires* (IRaMuTeQ), para realizar a análise lexical das evocações e das entrevistas. Além disso, empregou-se o Método de Reinert para analisar as entrevistas. Os sujeitos foram codificados por número (paciente 001, paciente 002 e assim sucessivamente), sexo (feminino e masculino), idade (menores ou igual a 52 anos e para maiores de 52 anos). Adicionalmente, as palavras que foram mais significativas (p < 0,0001) para cada classe distribuída pelo dendrograma da Classificação Hierárquica Descendente (CHD) serão descritas e sinalizadas com o valor da frequência na classe (f ) e qui-quadrado (x^2^). Esse procedimento visa fornecer uma compreensão mais aprofundada dos resultados obtidos. As Unidades de Contexto Elementar (UCEs) são segmentos textuais capturados do material de texto. Através delas, é possível compreender os discursos elaborados pelos sujeitos diante dos questionamentos realizados neste estudo^(19)^.

## RESULTADOS

A caracterização sociodemográfica dos 126 participantes demonstra que a maioria são do sexo masculino (n=74, 58,73%). A faixa etária predominante é de maiores de 52 anos (n=68, 53,97%). Quanto ao estado civil, metade declarou estar casado (n=63, 50%). Quanto ao nível escolar, menos da metade possui ensino médio completo (n=48, 38,10%), enquanto que outro grande grupo possui o ensino fundamental incompleto (n=35, 27,78%). Majoritariamente, os participantes residem na Região Metropolitana do Rio de Janeiro (n=100, 79,37%). Sobre a religião, a maioria declarou seguir a religião católica e a evangélica (n=52, 41,27%, respectivamente). A maioria possui diagnóstico de câncer há menos de um ano (n=59, 46,83%), e mais da metade não fez tratamento oncológico anteriormente (n=67, 53,17%). Em relação ao histórico familiar de câncer, grande parte afirma ter tido casos de câncer em algum parente, independentemente do grau de parentesco (n=85, 67,46%).

De acordo com os resultados das evocações livres de todos os participantes, com auxílio do *software* IRaMuTeQ, foram adotados os seguintes parâmetros: uma frequência mínima de palavras de 5, o que significa que os termos com frequência inferior a esse valor foram excluídos da análise; uma frequência média de 13,26; e uma média das ordens médias de evocação (OMEs) igual a 2,7. A OME está relacionada à média de posição de cada termo evocado no *corpus* analisado, indicando que, quanto menor o valor da OME, mais prontamente foi evocado pelos sujeitos em primeira instância ao elemento indutor “cirurgia”. A partir dos referidos parâmetros, o *software* gerou o Quadro de Quatro Casas, apresentado no Quadro 1, com os conteúdos e sua organização.

**Quadro 1:** Quadro de Quatro Casas ao termo indutor“cirurgia”para pessoas diagnosticadas com câncer atendidas em um Centro de Alta Complexidade em Oncologia, Rio de Janeiro, Rio de Janeiro, Brasil, 2022 (N=126)

OME	≤ 2,7			>2,7		
Freq. Med.	Termo evocado	Freq.	OME	Termo evocado	Freq.	OME
≥13,26	Medo	47	2,1			
	Cura	29	2,7	Tratamento	17	2,9
	Esperança	19	2,7	Anestesia	16	3,1
	Retira a doença	16	2,1			
< 13,26				Deus	12	3,4
	Dor	13	2,5	Cicatriz	11	3,2
	Corte	12	2,3	Nervoso	11	3,0
	Necessária	10	1,8	Tranquilo	11	2,9
	Ficar bem	9	2,3	Médico	10	3,0
	Saúde	9	2,7	Recuperação	9	3,1
	Ansiedade	7	2,7	Boa	9	3,3
	Melhora	6	2,5	Vida	8	3,5
				Pavor	8	3,1
				Confiança	6	3,8

*OME – ordem média de evocação.*

Observa-se neste estudo que o possível NC é formado por “medo”, “cura”, “esperança” e “retira a doença”. O léxico “medo” foi o termo mais evocado (47) e mais prontamente falado pelos sujeitos (OME= 2,1) ao termo indutor “cirurgia”, e isso reflete o sentimento negativo que os pacientes sentem diante do tratamento cirúrgico para o câncer. A“cura*”* foi o segundo termo mais evocado (29) pelos sujeitos, com OME 2,7, ou seja, os participantes veem a cirurgia como um procedimento que irá trazer a cura do câncer. É um elemento com teor positivo e que gera motivação e esperança pela realização do procedimento.

A “esperança” é o terceiro elemento mais evocado (19), com OME 2,7, reforçando o desejo pela cura, pois, apesar do medo, possuem esperança que, através da cirurgia, ficarão livres do câncer. Tal termo remete à dimensão imagética do tratamento cirúrgico como modificador da vida. Por fim, a expressão “retira a doença” é o elemento que possui a menor frequência (16) de evocação do quadrante, porém tem menor OME (2,1), posto que a cirurgia retira a doença e, consequentemente, a cura da doença. É um elemento representativo atitudinal que reflete o resultado da cirurgia.

A inclusão dos termos “cura”e “retira a doença”no NC revela as RS complexas dos participantes sobre o tratamento cirúrgico para o câncer.“Cura”denota a esperança profunda de uma recuperação completa e a eliminação definitiva da doença, refletindo a busca por um resultado positivo e duradouro. Por outro lado, “retira a doença”ressalta a percepção da cirurgia como um processo prático e concreto de remover fisicamente o câncer do corpo, resultando, assim, na erradicação da enfermidade. Esses dois termos, embora diferentes em sua conotação, compartilham uma meta comum de superar o câncer, representando tanto a dimensão simbólica da esperança quanto a dimensão pragmática da intervenção cirúrgica. A inclusão de ambos no NC do estudo ilustra a multiplicidade de significados atribuídos à cirurgia oncológica pelos pacientes, mostrando a complexidade e a riqueza das suas RS sobre o tema.

A primeira periferia é formada por “tratamento” e “anestesia”. São elementos ligados à proposta terapêutica cirúrgica e que são provenientes da dimensão cognitiva do que é cirurgia e seus instrumentos de realização, posto que, para os pacientes, a anestesia é uma das etapas do tratamento cirúrgico. São elementos com alta frequência de evocação, porém alta OME, o que justifica não participarem do possível NC da representação.

A segunda periferia é constituída pelos elementos “Deus”, “cicatriz”,“nervoso”,“tranquilo”,“médico”,“recuperação”,“boa”,“vida”, “pavor” e “confiança”. O termo “Deus” foi o mais evocado dentro desse quadrante, representando o apoio espiritual. A tríade“cicatriz”,“nervoso”,“tranquilo”representou os segundos termos mais evocados, enquanto que “tranquilo” teve a menor OME dentro da periferia, sendo elementos de dimensão cognitiva e afetiva/ atitudinal. O termo “médico” simboliza o profissional que está à frente do tratamento e veículo de concretização de processo. Os termos“recuperação”e“vida”pertencem ao campo da dimensão imagética, enquanto que “boa”, “pavor” e “confiança” pertencem à dimensão afetiva/atitudinal, sendo que “confiança”foi o termo com menor frequência de evocação e maior OME.

A ZC deste estudo é formada pelos elementos “dor”, “corte”, “necessária”,“ficar bem”,“saúde”,“ansiedade”e“melhora”. Os termos presentes nesse quadrante reforçam a ideia presente no NC, uma vez que o medo reflete a ansiedade, a dor, o corte e a cicatriz da realização do procedimento. Todavia, o termo“necessária”possui menor OME (1,8) de todo o quadro, ou seja, os pacientes entendem que a cirurgia é um tratamento para ficarem curados do câncer e eficaz na recuperação da saúde (“melhora”) e no ficar bem.

De acordo com a análise das entrevistas, através do Método de Reinert, originou-se o dendrograma da CHD, com seis classes, a partir dos 726 segmentos de texto, com 92,13% de aproveitamento do *corpus* analisado. Inicialmente, ocorreu uma primeira divisão, em que foi formado um eixo com a classe 6 (16,2%) e outro eixo com as outras classes segmentares. Em uma segunda divisão, foi formado um eixo com as classes 3 (13,8%) e 2 (13,9%), de um lado, e outro eixo com a classe 5 (12,5%), de outro lado. Em uma terceira divisão, foram geradas as classes 1 (22,7%) e 4 (20,8%), conforme mostra a Figura 1.

**Figura 1:** Dendrograma de Classificação Hierárquica Descendente por conteúdo semântico, Rio de Janeiro, Rio de Janeiro, Brasil, 2022 (n= 60)

A partir da análise das classes supramencionadas, foram identificadas seis classes, que serão apresentadas a seguir.

### Classe 1 – Impacto do diagnóstico de câncer e do tratamento cirúrgico

A classe 1 é a maior do *corpus*, formada por 165 UCEs, correspondendo a 22,7 % do *corpus*. Os elementos que tiveram maiores destaques nesta classe são ficar (f=71, x^2^=47,5), cabeça (f=19, x^2^=38,93), medo (f=25, x^2^= 23,95), doente (f=8, x^2^=22,71), perder (f=10, x^2^=19,28), conversar (f=8, x^2^=18,94) e triste (f=9, x^2^=16,3).

Os elementos refletem o impacto do diagnóstico e da cirurgia na vida dos pacientes, além dos sentimentos que experimentam neste momento. O receio do desconhecido, do que pode acontecer na cirurgia e da invasão que demanda o procedimento soa como uma avalanche de sentimentos no paciente. Cabe destacar também que a dimensão imagética do tratamento algumas vezes pode não corresponder ao resultado esperado do tratamento, o que gera também tristeza e frustração.

*Vou falar a verdade, senti medo. [...] medo, ansiedade, nervosismo, é uma mistura de sentimento que a gente não consegue explicar certo o que é não*. (Paciente 034, masculino, idade 52 anos ou mais)

*Cirurgia, para mim, é medo. A gente sente medo, ao mesmo tempo a gente sente que é obrigada a fazer, porque é um processo de cura e esperança.* (Paciente 036, feminino, idade 52 anos ou mais)

*A cirurgia, pra mim, foi uma esperança, mas antes eu estava com muita fé, muita esperança, mas depois que eu fiz, eu fiquei muito deprimida, fiquei muito frustrada, porque não é aquilo que eu imaginei na minha cabeça* [...]. (Paciente 046, feminino, idade 52 anos ou mais)

### Classe 2 – Hábitos de vida e rotina social dos participantes antes do diagnóstico de câncer

A classe 2 é formada por 101 UCEs, correspondendo a 13,9% de todo o *corpus*. Os termos que tiveram maiores destaques são casa (f=27, x^2^= 88,06), rua (f=11, x^2^=69,12), trabalhar (f=28, x^2^= 55,27) e sair (f=19, x^2^= 50,05). Tal grupo de palavras corresponde aos hábitos de vida e rotina social dos participantes antes do diagnóstico de câncer. Os termos brincar, criança e rua remetem às lembranças e à vida social do passado, enquanto que cerveja, fumar, trabalhar e sair remetem aos hábitos cotidianos.

*Antes do diagnóstico do câncer, a minha vida era desregrada. Fumava, bebia, fazia de tudo. Ficava à noite acordado, madrugada, viajava demais, trabalhava demais, tudo, tudo demais.* (Paciente 018, masculino, idade 52 anos ou mais)

*Era uma vida normal. Brincava na rua, até depois que eu recebi o diagnóstico, fiz as coisas, eu tentei levar uma vida normal,* [...], *jogar uma bola, brincar igual criança,* [...]. *Essas coisas, assim, eu tive que retroceder um pouco, parar um pouco.* (Paciente 024, masculino, idade menor ou igual a 52 anos)

### Classe 3 – Práticas do cotidiano e sua relação com o tratamento cirúrgico: ressignificando a nova realidade

A classe 3 é construída por 100 UCEs, referindo-se a 13,8% do *corpus* analisado. As palavras que se destacam na classe são normal (f=43, x^2^= 114,14), voltar (f=24, x^2^=54,84), esperar (f=21, x^2^= 45,46), antes (f=22, x^2^=32,09) e vida (f=45, x^2^=28,64). Nota-se a paridade bem próxima com a classe 2, porém essa faz menção às práticas do cotidiano e aos desejos de retomar à normalidade do dia a dia, como era antes de passar pelo procedimento e do diagnóstico, ressaltando ainda o desejo de ser independente, faceta mascarada após passarem pelo processo cirúrgico.

*Saúde, vida, a cirurgia me trouxe saúde e me trouxe vida.* (Paciente 013, feminino, idade 52 anos ou mais)

*Que eu possa trabalhar, viajar para ver minha família, cuidar da minha casa, da minha vida como era antes, não depender dos outros para estar fazendo as coisas para mim.* (Paciente 015, feminino, idade menor ou igual a 52 anos)

*E com a cirurgia, vai resolver muita coisa na minha vida, vai me dar mais disposição. Vai me levar de volta ao mercado de trabalho. Isso também estava me impedindo de voltar a trabalhar*. (Paciente 032, feminino, idade menor ou igual a 52 anos)

Nota-se a necessidade dos pacientes em se readaptar à nova condição de vida, de forma que aproxime o mais próximo da sua antiga normalidade. O reajuste social e o anseio pela recuperação ajudam a mantê-los mais otimistas diante da nova condição de vida.

### Classe 4 – Dimensão prática da cirurgia, a rotina de tratamento oncológico e a dimensão espiritual

A classe 4 é composta por 151 UCEs, referindo-se a 20,8% do *corpus*. Os termos que se destacam nessa classe são Deus (f=59, x^2^=89,84), graça (f=15, x^2^=36,68), mão (f=13, x^2^=29,63), aqui (f=40, x^2^=25,96), hospital (f=9, x^2^=18,85), experiência (f=4, x^2^=15,32) e permissão (f=4, x^2^=15,32).

De acordo com os elementos que configuram essa classe, destaca-se, nos discursos dos pacientes, a dimensão prática da cirurgia por meio de cognemas, que expressam a rotina do tratamento oncológico e a dimensão espiritual nesse novo cenário que se apresenta. Os termos espelham os sentimentos presentes no ato de internação, que se traduz nos momentos do pré-, trans e pós-operatório imediato. Por outro lado, existem elementos que refletem a fé em um Ser que transcende os planos carnais, como Deus.

*Tudo que acontecer vai ser vontade de Deus, permissão de Deus.* (Paciente 007, masculino, idade menor ou igual a 52 anos)

[...] *o primeiro contato que eu tive com o doutor, eu senti a presença de Deus nele.* (Paciente 035, feminino, idade menor ou igual a 52 anos)

### Classe 5 – Sentimentos e anseios diante da cirurgia: a cura oncológica

A classe 5 é a menor do *corpus*, configurada por 91 UCEs, representando 12,5% do *corpus*. Tal conjunto reflete o sentimento e o desejo depositados no sucesso do tratamento cirúrgico para seguir a vida livre de doença, como pode ser visto por meio dos elementos cura (f=21, x^2^=103,65), vitória (f=7, x^2^=49,32), livre (f=7, x^2^=41,47), doença (f=19, x^2^=40,13), recomeço (f=5, x^2^=35,13), esperança (f=12, x^2^=31,78) e salvação (f=4, x^2^=28,07).

*É a possibilidade de eu ficar livre do câncer e voltar à vida normal. A cura.* (Paciente 053, masculino, idade 52 anos ou mais)

*A cirurgia no meu caso foi salvação, para mim foi a salvação, um recomeço e uma grande vitória.* (Paciente 013, feminino, idade 52 anos ou mais)

*Representa me livrar de um problema, representa vitória.* (Paciente 049, masculino, idade menor ou igual a 52 anos)

A partir do discurso das entrevistas, é possível contextualizar os elementos presentes no NC da representação, no qual a RS da cirurgia para os pacientes oncológicos gira em torno da esperança de tirar a doença e, consequentemente, ficarem curados através do tratamento cirúrgico, ao ponto que sentem medo da cirurgia e dos percalços que podem vir no pós-operatório.

### Classe 6 – Rede de apoio do paciente diante da cirurgia oncológica

A classe 6 é composta por 118 UCEs, representando 16,2% do *corpus*, tendo como principais palavras apoio (f=37, x^2^=163,57), religioso (f=23, x^2^=122,39), receber (f=29, x^2^=106,27), família (f=36, x^2^=105,83) e financeiro (f=20, x^2^=99,12). Analisando o conteúdo lexical das entrevistas que formam a classe 6, percebe-se que esta reflete o apoio social, espiritual, mental e financeiro que os pacientes recebem diante da cirurgia. Essas características são percebidas através dos discursos a seguir:

*Da minha família, sim, muito apoio, minha família foi tudo, foi a minha base e amigos também.* (Paciente 003, masculino, idade menor ou igual a 52 anos)

*Eu recebi muito apoio da minha família, muitos amigos. A psicóloga daqui também me ajudou muito. Os médicos também me deram suporte legal.* (Paciente 006, feminino, idade menor ou igual a 52 anos)

Observa-se que os laços sociais, espirituais e profissionais retroalimentam as bases que sustentaram o tratamento cirúrgico, como pode ser confirmado através também da análise prototípica (Quadro 1). Ressalta-se a importância da presença da tríade Deus-família-profissional para suportar o diagnóstico ameaçador da vida, garantir a confiança no tratamento proposto e galgar sentimentos positivos diante das alterações provocadas pela doença.

## DISCUSSÃO

As RS da cirurgia oncológica para pacientes com câncer se organizam ao redor de três dimensões: a prática, que inclui os hábitos e ações antes do diagnóstico e em relação à cirurgia; a afetiva, retratada aqui em um contínuo com dois polos, com o primeiro sendo o medo e, o segundo, no outro extremo, sendo a esperança; e a imagética, em que cura, retira da doença e tratamento se inter-relacionam, formando, em conjunto, uma imagem da cirurgia oncológica que se consubstancia em uma intervenção específica que resolve alterações pontuais e bem delimitadas.

Destaca-se que a anestesia também se insere na dimensão imagética, em função de uma ambiguidade de sua construção simbólica. Por um lado, ela é imprescindível para a realização da cirurgia, mas, por outro, está presente em conversas cotidianas que contêm histórias e experiências negativas relacionadas a ela, como a morte após ser sedado (dormir e não mais acordar) e sequelas como consequências de seu uso. Torna-se importante apontar, no conjunto dos resultados, a identidade social de grupo que se mostra na presença da rede de apoio, assim como um aspecto relacional dos pacientes com a dimensão do sagrado.

O medo, a ansiedade e o nervosismo são sentimentos muito presentes nos pacientes, devido a cirurgia ser um procedimento invasivo e que demanda muitos cuidados no prée pós-operatório^(20,21)^. A hospitalização é muitas vezes um cenário novo e que gera muitos sentimentos negativos, bem como a falta de informação ou incompreensão de como será feita a cirurgia^(22)^. Estudo^(23)^ realizado com 25 pacientes submetidos à cirurgia mostra que a ansiedade é um sentimento muito aflorado nos pacientes, o qual os fazem experimentar sensações de formigamento, medo do que pode acontecer e da morte, nervosismo, entre outros.

A ansiedade antecipatória é uma preocupação mais clínica do que propriamente do ato cirúrgico. Essas reações emocionais sugerem que os indivíduos constroem representações mentais coletivas sobre eventos e situações, influenciadas por fatores sociais e culturais. Pesquisa realizada com 3.087 pacientes cirúrgicos submetidos a qualquer tipo de anestesia e cirurgia aponta que 40% dos pacientes demonstraram-se ansiosos no período pré-operatório^(24)^. Outra pesquisa com 109 pacientes submetidos à biópsia de tumor cerebral ou craniotomia com ressecção do tumor destacou que 30% dos pacientes apresentavam níveis clínicos de ansiedade antes do procedimento, em comparação com 20% em um grupo de pacientes submetidos à cirurgia eletiva da coluna^(25)^.

Outra experiência vivenciada pelos pacientes são as modificações dos segmentos corporais provocadas pelas cirurgias, que geram muitas perdas, entre elas a autoestima, o convívio social e a autonomia. Logo, percebe-se que a terapêutica agride o paciente em diversos aspectos físicos, sociais e mentais^(26,27,28)^. De acordo com a pesquisa de Peixoto *et al*.^(29)^ com 56 pacientes com estomia em pós-operatório tardio, evidenciou-se que 48,2% dos participantes apresentaram complicações relacionadas à estomia. Além disso, de acordo com a avaliação de adaptação à ostomia, no domínio aceitação positiva, as pessoas tiveram uma pontuação média, ou seja, a maioria não se adaptou à estomia. Isso traduz o medo, a cicatriz, o corte, o conhecimento reificado do paciente diante da cirurgia, bem como o descontentamento pós-cirurgia explicitado pela participante 046, na classe 1, da análise da CHD.

Estudo realizado com 41 pacientes diagnosticados com neoplasia de cavidade oral revelou que mais de 80% dos pacientes receberam tratamentos de cirurgia, sendo que, após a exérese do tumor, os pacientes apresentaram deformidades permanentes, com impacto diretamente na qualidade de vida global (QVG). O sexo masculino apresentou a pior QVG na esfera social, enquanto que o sexo feminino apresentou a pior QVG na escala física^(30)^. Além disso, o mesmo estudo ressalta que a palavra“medo”esteve ligada aos termos“triste”e“morrer”, refletindo, assim, a consciência de uma doença ameaçadora da vida^(30)^, mas, neste estudo, o medo estava mais atrelado ao procedimento propriamente do que ao diagnóstico de câncer.

A cirurgia é vista como uma forma de tratamento para os pacientes e, através dela, é possível alcançar a cura. Na obra de Antunes *et al*.^(20)^ com 111 participantes, o termo“tratamento”está presente no possível NC da representação, enquanto que, neste estudo, o mesmo elemento encontra-se na 1º periferia, ou seja, isso reforça que, em algum momento, esse termo será elemento central da representação, reforçando a dimensão cognitiva a respeito da cirurgia. Outrossim, o termo “cura” encontra-se no possível NC da representação, reafirmando, assim, o tratamento como intermédio da cura.

Estudo demonstrou que os pacientes veem o câncer como uma doença normal e difícil, porém acreditam no tratamento e na cura através dos recursos disponíveis, mesmo sabendo que será um processo difícil. Além disso, os resultados apontam que a cura será alcançada pela intervenção de Deus e do tratamento, associados ou não^(31)^. Pesquisa sobre a RS do câncer revelou que o elemento “esperança” foi o mais prontamente evocado pelos sujeitos na segunda periferia, refletindo, assim, a confiança e o otimismo pelo tratamento em busca da cura do câncer^(31)^.

A espiritualidade/religiosidade é um mecanismo importante de enfrentamento diante do procedimento cirúrgico^(32)^. Na segunda periferia, ressalta-se a presença da tríade Deus, médico e confiança. Este cenário centraliza-se no caminho rumo à cura do câncer através de duas vertentes que não se excluem mutuamente: o conhecimento científico representado pelo médico; e a fé em uma entidade superior, manifestada pela religiosidade (Deus). Pesquisas apontam que o momento do diagnóstico oncológico tem sido um dos momentos mais traumáticos do ponto de vista psicológico e existencial dos pacientes e familiares, sendo tal dimensão basilar no enfrentamento da patologia, em que se tem a fé como eixo central e norteador do processo de recuperação, de esperança e de cura da doença. Ademais, a religiosidade é recurso fundamental diante da hospitalização e adoecimento, tornando-se ferramenta importante para lidar com as dores, angústias, medos e outros sentimentos negativos atrelados a esse processo^(22,32,33,34)^.

Além do apoio espiritual, os pacientes contam com o apoio familiar durante o diagnóstico de câncer, revelando, assim, uma ferramenta fundamental e persistente em todo o processo de tratamento da doença. No estudo de Wakiuchi *et al*.^(31)^, esse apoio aparece através do termo “família”, na segunda periferia. Neste estudo, o apoio é visto através da análise da CHD, na classe 6. Estudo de Fischer e Seibaek^(35)^ revela que, além do apoio familiar, o grupo social, como vizinhos e pessoas adoecidas pela mesma doença, mostrou-se um mecanismo valioso para os pacientes.

Alguns pacientes referiram o desejo de voltar à normalidade. Em outras palavras, esse sentimento expressa a vontade de viver o mais próximo do que vivia antes de passar pelo procedimento cirúrgico, como trabalhar, não ter muitas restrições no modo de viver, ressignificar alguns momentos não vividos. Em estudo sobre a experiência de pessoas que vivem com câncer em estágio avançado, os autores afirmam que a “normalidade” é o ajuste à nova realidade, correspondendo à vida que se tinha antes de possuir a doença, bem como desenvolvendo uma nova relação com o ser finito e a morte^(36)^.

O tratamento oncológico gera grandes modificações no cotidiano dos portadores da doença, como afastamento do mercado de trabalho, da vida social e do sentir-se ativo. Revisão sistemática com metanálise de estudos observacionais^(37)^ identificou que a cirurgia de câncer de mama está associada a maiores taxas de desemprego, sendo que 26 estudos (n= 46.927 pacientes) fizeram associação de 127 variáveis com o desemprego após o procedimento. Entre os fatores que predisponham ao afastamento do mercado de trabalho, destacam-se as altas demandas psicológicas e físicas de trabalho, associadas a outras demandas após a cirurgia, como reabilitação, associação da quimioterapia e da radioterapia, as idas constantes ao hospital e aos próprios cuidados que demandam a mastectomia, gerando suporte de emprego ou acomodação em horário de trabalho flexível, licença remunerada e/ou modificações de tarefas de trabalho^(37)^.

### Limitações do estudo

Uma limitação do estudo é o fato de alguns pacientes terem sido abordados no período pós-operatório imediato e mediato. Tais condições afetam a percepção do viver após a cirurgia no ambiente extra-hospitalar, visto que ainda estavam internados e sob cuidados da equipe de enfermagem. Nesse contexto, surge a possibilidade de novos estudos que atendam às representações da cirurgia no pós-operatório tardio, em que o paciente já teve contato com a sua realidade no cotidiano.

### Contribuições para as áreas da enfermagem, saúde ou políticas públicas

Destarte, acredita-se que, através deste estudo, os profissionais de saúde poderão embasar seus cuidados assistenciais para atender às demandas e representações elencadas pelos sujeitos diante de tal procedimento, com vistas à redução do estresse causado pelo procedimento. Acredita-se ainda na inovação e na promoção do cuidado através dos resultados desta pesquisa, ao entender e conhecer as RS da cirurgia para os pacientes, pois, ao se desvelar tais representações, será possível acessar e explorar as atitudes, os valores, os pensamentos e as práticas de como o grupo se comporta diante da doença e do tratamento, promovendo um cuidado mais holístico e humanizado.

## CONSIDERAÇÕES FINAIS

Os sentimentos diante da cirurgia são ambivalentes, devido ao tempo que os pacientes sentem aflição, pavor da cirurgia e medo dos percalços pós-procedimento. Adicionalmente, identifica-se a confiança em Deus e no médico; com isso, alguns usuários ficam calmos e confiantes, sabendo que a cirurgia será otimista, possibilitando a cura ou ausência de doença.

A partir da análise prototípica e dos discursos, é possível descrever uma conjuntura a respeito da importância da cura enquanto resultado esperado após a cirurgia, avaliada predominantemente como retirada da doença. São elementos com teor positivo, exceto “medo”, que representa fatores negativos da cirurgia. A partir dos conteúdos do possível NC, é possível identificar a presença de dimensões afetiva-atitudinal e cognitiva que formam as RS.

Sendo assim, a atuação multidisciplinar às pessoas vivendo com câncer em vigência de tratamento cirúrgico necessita ser revista, considerando a RS do grupo onde se tem o termo“medo” com a maior frequência das evocações, além da ideia de cura a partir da remoção da doença. À vista disso, as consultas pré-operatórias são de extrema importância, a fim de esclarecer as dúvidas existentes acerca do procedimento, bem como a oferta de acolhimento ao paciente para minimização de sentimentos negativos sobre o procedimento.
